# Development and evaluation of an augmented reality serious game to enhance 21st century skills in cultural tourism

**DOI:** 10.1038/s41598-025-95615-5

**Published:** 2025-04-18

**Authors:** Pawitra Liamruk, Natnicha Onwong, Kiettikoon Amornrat, Ann Arayapipatkul, Kawin Sipiyaruk

**Affiliations:** 1 Faculty of Information and Communication Technology, Mahidol University, Nakhon Pathom, Thailand; 2 Department of Orthodontics, Faculty of Dentistry, Mahidol University, Bangkok, Thailand

**Keywords:** 21st century skills, Augmented reality, Cultural tourism, Serious game, Educational technology, Game-based learning, Socioeconomic scenarios, Environmental social sciences, Sustainability

## Abstract

Cultural tourism plays a significant role in preserving traditions and promoting global awareness. However, engaging younger generations with cultural heritage through traditional tourism methods remains challenging, as traditional tourism methods are often perceived as lacking the interactivity and engagement characteristic of digital experiences. The increasing prevalence of digital entertainment and social media has further shifted preferences toward dynamic, personalized content. Integrating modern technologies, such as augmented reality (AR), into cultural tourism experiences can bridge this gap, providing interactive and immersive learning opportunities. This research aimed to develop and assess the usability and educational impact of KideClass in enhancing 21st century skills while promoting cultural tourism. A descriptive evaluation design was employed, with 20 participants: 10 children (aged 6–12 years) and 10 parents. Participants engaged in six mini-games integrated into the KideClass game, designed to enhance cultural and scientific literacy. Data were collected using perceived evaluations of 21st century skill development and a user satisfaction assessment based on a 5-point Likert scale. The results indicated perceived improvements in 21st century skills, particularly in critical thinking/problem-solving (Children: Mean = 4.57, SD = 0.45; Parents: Mean = 4.30, SD = 0.33) and adaptability (Children: Mean = 4.47, SD = 0.55; Parents: Mean = 4.17, SD = 0.32). Children reported higher ratings in scientific literacy (Mean = 4.47, SD = 0.48) compared to parents (Mean = 3.83, SD = 0.36). User satisfaction was high, with audio esthetics (Mean = 4.90, SD = 0.32), creative freedom (Mean = 4.65, SD = 0.67), and AR features (Mean = 4.65 ± 0.78) receiving top ratings. However, usability (Mean = 4.43, SD = 0.69) and narrative clarity (Mean = 4.30, SD = 0.74) were identified as lower than other aspects. Overall, KideClass demonstrates significant potential in enhancing cultural tourism engagement and developing key 21st century skills among children and parents. The integration of AR and gamified learning provides a dynamic and interactive platform for cultural education. While the game was well perceived in terms of user engagement and satisfaction, further refinement in usability and narrative clarity is recommended. Future iterations of the game could benefit from expanded cultural scenarios and collaborative features to foster deeper learning and engagement.

**Supplementary Information:**

The online version contains supplementary material available at 10.1038/s41598-025-95615-5.

## Introduction

Cultural tourism plays a vital role in preserving traditions, fostering cross-cultural understanding, and supporting local economies. With the rich heritage of communities, it not only celebrates unique identities but also promotes global awareness and appreciation^1^. Moreover, cultural tourism provides opportunities for sustainable development by creating jobs and generating income for local artisans, performers, and cultural practitioners^2,3^. Its value extends beyond economic benefits, as it serves as a platform for education and dialogue, allowing people to connect with history, art, and traditions^4^. However, ensuring the relevance and sustainability of cultural tourism in a rapidly changing world is challenging. In particular, the ability to engage and inspire younger generations is critical for securing the future of cultural preservation^5,6^. This leads to the question of how to adapt cultural tourism strategies to meet the preferences and expectations of modern audiences.

Despite its significance, cultural tourism struggles to capture the attention of younger generations, who are often disengaged from traditional cultural presentations. Unlike older audiences who may appreciate historical sites or museum displays, digital natives prefer dynamic and interactive experiences^7^. There is a number of studies reporting younger generations’ disengagement with cultural tourism in several countries^8–10^. Static exhibitions, brochures, and conventional tours fail to meet their expectations for engagement, leading to a widening gap between cultural heritage and the interests of modern audiences^11,12^. This disengagement stems from the lack of storytelling, interactivity, and technology-driven content that aligns with the habits of younger users. The evidence emphasizes the need for innovative approaches to make cultural tourism appealing and accessible, especially for tech-savvy individuals who learn and interact through digital media^13,14^. Bridging this divide requires reimagining cultural experiences to incorporate elements of play, exploration, and personalization. Addressing these challenges lays the foundation for employing gamified learning, an approach that has proven successful in other educational contexts.

Game-based learning has revolutionized education by integrating game mechanics such as rewards, challenges, and feedback into teaching methods. This approach transforms passive learning into an engaging and active process, motivating learners to explore and solve problems in an enjoyable way^15–17^. A number of studies show that game-based learning can improve critical thinking, collaboration, and retention by immersing participants in real-world scenarios^18–20^. In cultural contexts, gamified experiences can make abstract concepts more tangible, turning history and tradition into interactive stories. By applying these principles to cultural tourism, game-based learning can foster a sense of curiosity and exploration, encouraging users to connect with cultural heritage in meaningful ways^21^. The synergy between gamified learning and modern technology, particularly Augmented Reality (AR), creates even greater potential for enhancing cultural engagement.

AR technologies offer opportunities to merge game-based learning with cultural tourism, creating immersive and interactive experiences that appeal to younger audiences. By overlaying digital elements onto real-world settings, AR enables users to engage with cultural heritage in ways that are both entertaining and educational^22,23^. Through features such as virtual reconstructions, interactive storytelling, and gamified challenges, AR can bring historical sites and traditions to life, transforming passive observation into active participation^24^. Unlike traditional methods, AR fosters exploration and discovery, allowing users to interact with their surroundings dynamically. Integrating AR into cultural tourism can bridge the gap between the physical and digital worlds, ensuring that cultural heritage remains relevant and captivating.

According to the challenges in engaging younger generations with cultural heritage, this research developed an AR serious game for cultural tourism, using Mahasawat Community as a case study. By integrating augmented reality and gamification, the game aims to bridge the gap between traditional cultural experiences and modern digital engagement, making cultural learning accessible, immersive, and appealing to tech-savvy audiences. The aim of this research was to evaluate the usability and educational impact of the AR serious game, focusing on its ability to promote 21st century skills while deepening appreciation for cultural heritage. To achieve this, the study sought to examine the effectiveness of an AR serious game in enhancing engagement, fostering 21st century skills, and promoting cultural tourism.

## Materials and methods

### Research design

This study employed a descriptive evaluation design, a methodology commonly used in educational and intervention-based research to systematically assess the outcomes and experiences of participants without manipulation of variables. Descriptive evaluation focuses on collecting and analyzing data to describe the effectiveness, usability, and impact of an intervention on its intended outcomes, providing a nuanced understanding of its success in real-world contexts. This approach was selected to evaluate the usability and educational impact of an AR serious game in enhancing 21st century skills and promoting cultural tourism.

### KideClass: AR serious game for promoting cultural tourism

**Fig. 1:**
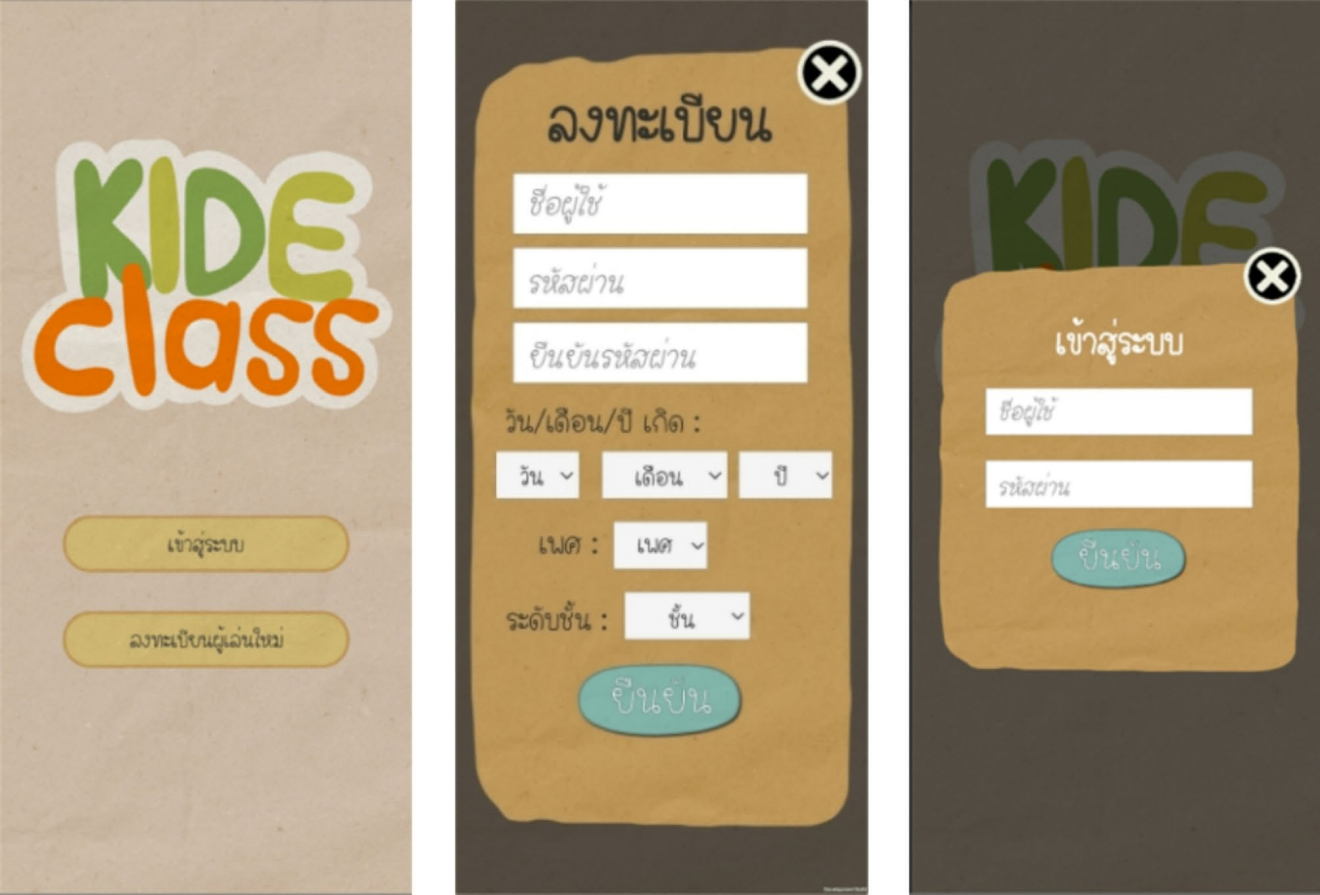
User interface of KideClass, showcasing log-in and registration screens, along with the main page layout before accessing the mini-games.

KideClass is an AR serious game developed to promote cultural tourism and enhance 21st century skills (Fig. 1). Through AR technology, the game offers an immersive and interactive experience themed around Mahasawat Community. Users register to track progress, scores, and achievements, creating a personalized and engaging experience. Built using the Unity engine and ARCore, KideClass incorporates Firebase Realtime Database to deliver real-time feedback and manage user data efficiently. By merging cultural exploration with skill-building, KideClass serves as a unique educational tool grounded in real-world interactions.

**Fig. 2:**
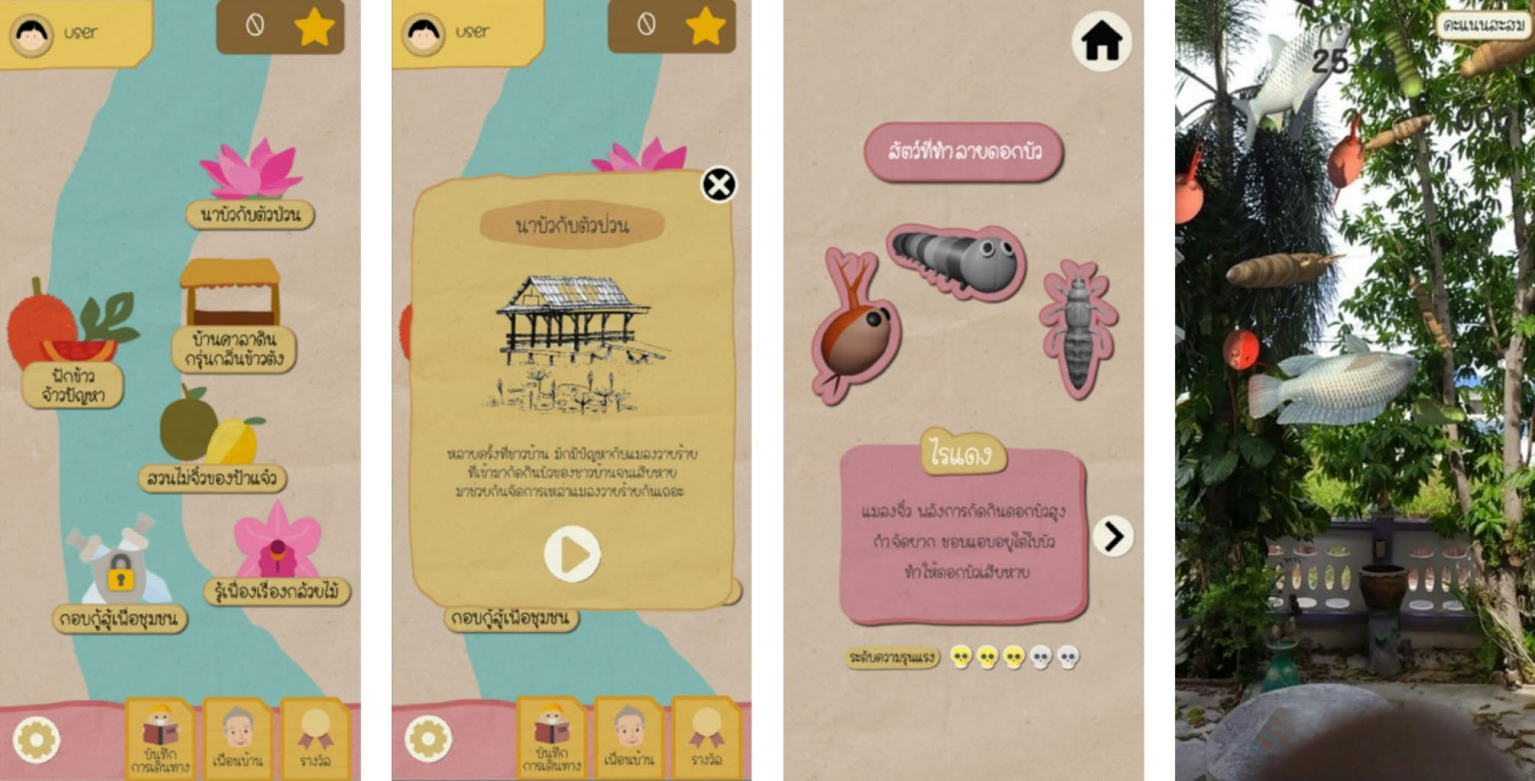
User interface of KideClass, presenting screenshots of a mini-game where users interact with AR elements.

KideClass consists of six mini-games, each designed to integrate AR technology with real-world cultural and scientific learning. These mini-games collectively foster 21st century skills such as critical thinking, creativity, adaptability, and financial literacy while enhancing engagement with cultural tourism (Fig. 2). Each game aligns with one or more core learning themes: scientific exploration, environmental sustainability, cultural heritage, and problem-solving. Detailed descriptions of all KideClass mini-games are provided in Supplementary Material 1.

**Fig. 3:**
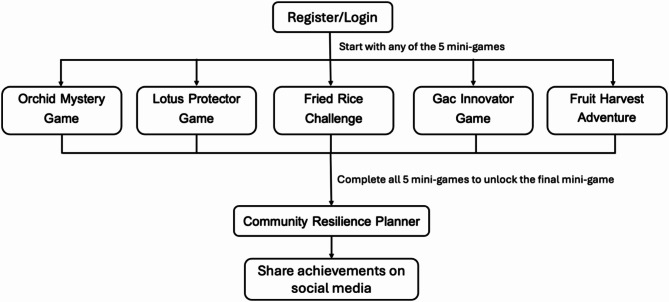
Diagram illustrating the game sequence and progression.

Upon registering or logging in, users can choose any of the five mini-games in no predetermined sequence. The game dynamically adapts based on the location they visit first, ensuring a non-linear experience that reflects real-world exploration. Once all five mini-games are completed, users unlock the final challenge, ‘Community Resilience Planner’. Finally, users can share their achievements on social media. The sequence of the gameplay is illustrated in Fig. 3.

#### Orchid mystery game

This game allows users to explore an AR-enhanced orchid farm, scanning QR codes to learn about orchid families, cultivation techniques, while answering related questions, such as ‘Which type of container is NOT suitable for planting orchids?‘. This fosters scientific literacy and curiosity.

#### Lotus protector game

This game immerses users in the ecological and cultural importance of lotus farming. Using AR, they must identify and manage pests, distinguishing between harmful and beneficial organisms. A timed matching activity reinforces the lotus life cycle, promoting environmental awareness and cultural literacy.

#### Fried rice challenge

This game challenges users to assist a non-player character in recreating a lost fried rice recipe. Users are asked to follow interactive steps, including kneading, rolling, drying, and frying rice, while making decisions that impact the dish’s final outcome. This game blends cultural appreciation with problem-solving and creativity in food presentation.

#### Gac innovator game

This game tasks users with transforming Gac fruit into innovative products, such as juice, soap, or lotion, with a focus on sustainability and entrepreneurship. Through experimentation and ingredient combinations, users enhance initiative, resourcefulness, and sustainable thinking, emphasizing the economic and environmental potential of local produce.

#### Fruit harvest adventure

This game tasks users with harvesting fruit using AR while engaging in numeracy challenges. Users must identify and collect specific fruits under time constraints and calculate prices to make correct change for virtual customers. This promotes numeracy, problem-solving skills, and financial literacy.

#### Community resilience planner

This game places users in a flood management simulation, where they allocate resources, plan crisis responses, and support community recovery. Users use stars earned in previous games as currency to purchase supplies for prevention and crisis management. This game strengthens financial literacy, leadership, and adaptability, while promoting social responsibility and strategic thinking.

**Fig. 4:**
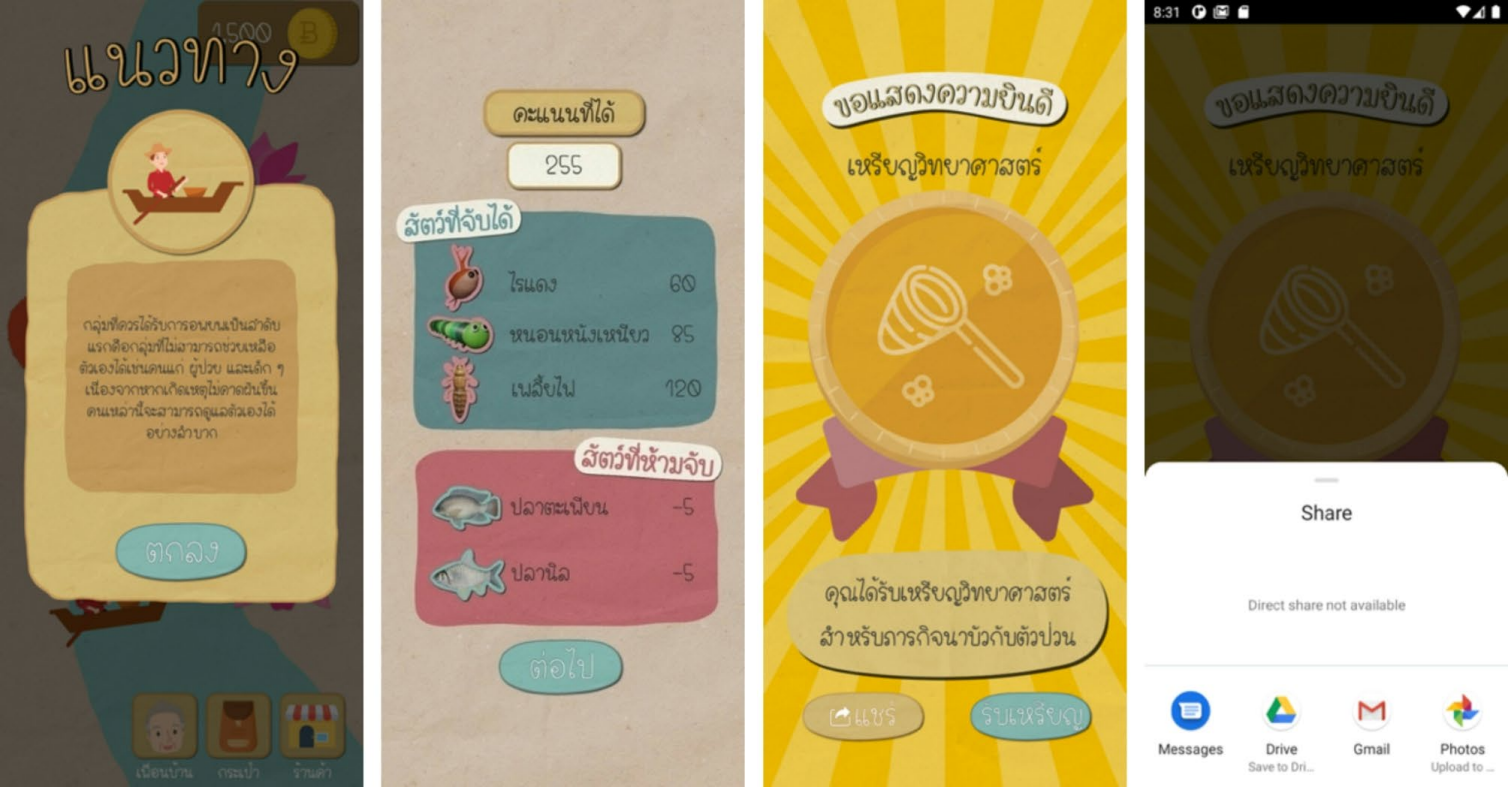
User interface of KideClass, presenting feedback, guidance, and achievements that users can share after completing tasks.

During gameplay, users receive real-time feedback through the game interface to guide their progress and help them successfully complete tasks (Fig. 4). This immediate feedback enables users to understand their performance and identify areas for improvement while navigating the challenges of each mini-game. At the end of each mini-game, users are presented with scores, providing a quantitative evaluation of their performance and reinforcing their achievements. Additionally, the interface allows users to share their accomplishments on social media, fostering a sense of motivation. By encouraging users to share their experiences online, the platform not only enhances user interaction but also promotes the community to a wider audience. These features make the gameplay experience both interactive and rewarding, aligning with best practices in educational game design, while while supporting the expected outcomes of the project in terms of enhancing cultural appreciation and tourism growth.

### Mahasawat community: setting for KideClass

The Mahasawat Community, located along the Mahasawat Canal in Nakhon Pathom Province, Thailand, serves as the central setting for the KideClass AR Serious Game. The selection of the Mahasawat Community as the setting for KideClass is driven by its rich cultural and agricultural heritage, alignment with the game’s learning objectives, potential for technological enhancement, accessibility, and contribution to sustainable tourism. With its agricultural heritage, the area features activities such as lotus farming, orchid cultivation, and traditional rice production. It is also part of Thailand’s agro-tourism initiatives, offering hands-on experiences like lotus picking, rice cracker making, and exploring fruit orchards.

KideClass integrates the cultural and agricultural essence of the Mahasawat Community to create mini-games that encourage critical thinking, creativity, and cultural awareness. Users engage with activities inspired by local traditions and daily life, such as sustainable farming practices and traditional crafts, all enhanced through augmented reality. By incorporating the heritage of the Mahasawat Community, the game promotes cultural tourism and educates users about the historical and cultural significance of this area.

### Research participants

The participants in this study consisted of primary school students (ages 6–12) and their parents, recruited using convenience sampling. The children represented the primary target audience for the intervention, while their parents participated as supporters, providing feedback and assisting during gameplay sessions. Although developmental differences exist within this age range, the study does not categorize participants into specific age groups. Instead, it evaluates overall engagement and skill development across all participants, recognizing that children interact with the game at varying levels based on their individual prior experiences and skills.

The sample size was expected to recruit ten pairs of participants, as they can uncover 80–95% of usability problems, making it sufficient for achieving the goals of this research^25^. To minimize bias associated with convenience sampling, strict inclusion and exclusion criteria were applied. Inclusion criteria required students to be aged 6 to 12 years and accompanied by parents who were willing to provide written informed consent and assist their child during the study. Exclusion criteria included students with learning difficulties, visual impairments, or other conditions that might interfere with gameplay, as well as participants unable to commit to the full duration of the study, which included orientation, gameplay, and feedback sessions.

### Data collection tools

#### Perceived evaluation of 21st century skill development

The perceived assessment tool, adapted from the Writer Self-Perception Scale (WSPS)^26^, was utilized to evaluate participants’ and parents’ perceptions of their development in 21st century skills after engaging with KideClass. This tool targeted 13 core competencies, including numeracy, scientific literacy, financial literacy, cultural literacy, critical thinking, creativity, communication, curiosity, initiative, persistence, adaptability, leadership, and social awareness (Supplementary Material 2). These skills were selected to align with the educational objectives of the game and the broader framework of 21st century skill development. Participants responded to 39 statements designed to reflect their experiences with these skills, with each skill assessed across three dimensions of self-perception:

**General progress (GPR):** Overall improvement in the skill.

**Specific progress (SPR):** Ability to apply the skill in specific tasks.

**Physiological states (PS):** Emotional responses associated with learning the skill.

Responses were collected on a 5-point Likert scale ranging from 5 (Strongly agree) to 1 (Strongly disagree). This tool provided valuable insights into how participants experienced the game as a platform for skill development, with the multi-dimensional framework enabling a comprehensive evaluation of learning outcomes.

#### User satisfaction assessment

The user satisfaction assessment for KideClass was adapted from the Game User Experience Satisfaction Scale (GUESS), ensuring a comprehensive evaluation of user satisfaction^27,28^. This tool was adapted to assess user satisfaction across specific dimensions relevant to both the gameplay and educational objectives of KideClass. The assessment comprised 20 items, organized into eight key aspects of game experience: ‘Usability’, ‘Narratives’, ‘Enjoyment’, ‘Creative freedom’, ‘AR features’, ‘Audio esthetics’, ‘Visual esthetics’, and ‘Personal gratification’ (Supplementary Material 3). ‘Play engrossment’ was excluded, as KideClass focuses on educational engagement and skill development rather than immersive gameplay, with its elements integrated into ‘Narratives’ and ‘Enjoyment’. ‘Social connectivity’ was excluded, as the game is primarily single-player, lacking multiplayer features or opportunities for collaboration. ‘AR features’ was separated from visual esthetics to evaluate unique interactive components like 3D model integration and usability. Each dimension was evaluated using a 5-point Likert scale ranging from 5 (Strongly agree) to 1 (Strongly disagree), where higher scores indicated greater satisfaction.

### Data analysis

Descriptive statistics were employed to analyze the data collected from the perceived evaluation of 21st century skill development and user satisfaction assessment of KideClass. For the perceived evaluation of 21st century skill development, participants’ responses across 13 competencies and three dimensions (GPR, SPR, and PS) were analyzed. The mean and standard deviation scores were used to evaluate the overall self-perceived development of 21st century skills among children and their parents. For the user satisfaction assessment, descriptive statistics summarized scores across eight aspects of system usability. Data for each aspect were calculated to identify strengths and areas for improvement in the game design.

### Ethical approval

This research was approved by the Mahidol University Central Institutional Review Board (COE No. MU-CIRB 2020/014.2801). All methods were performed in accordance with the relevant guidelines and regulations. Written informed consent was obtained from all participants and their parents prior to data collection.

## Results

### Research participants

This research included a total of 20 participants: 10 children (6 females and 4 males, aged 6–12 years) and 10 adults (9 females and 1 male, aged 27–60 years). The adult participants, related to the children as parents, aunts/uncles, or grandparents, served as observers and collaborators. This diverse participant pool enabled a comprehensive evaluation of the game’s usability and educational impact, encompassing both child and adult perspectives.

### Perceived evaluation of 21st century skill development

The self-assessment results, presented in Table 1, summarize the perceived development of 13 key competencies among children and their parents. ‘Critical thinking/Problem-solving’ received the highest ratings from both groups, though children rated themselves slightly higher (Mean = 4.57, SD = 0.45) than parents (Mean = 4.30, SD = 0.33). Notable generational differences emerged in ‘Scientific literacy’, where children rated themselves (Mean = 4.47, SD = 0.48), higher than their parents (Mean = 3.83, SD = 0.36), suggesting a stronger sense of confidence or engagement with scientific concepts among children. In contrast, ‘Financial literacy’ was rated higher by parents (Mean = 4.27, SD = 0.31) than by children (Mean = 3.87, SD = 0.69), indicating that parents may perceive financial competency as more developed than children acknowledge in themselves. Additionally, children provided higher ratings for ‘Creativity’ and ‘Leadership’, while parents rated ‘Persistence/Grit’ more favorably. Overall, children tended to rate themselves higher in skill acquisition compared to their parents. These findings highlight the generational differences in evaluating 21st century skills, emphasizing the role of self-perception in learning and skill development. More detailed results are provided in Supplementary Material 4.

**Table 1:** Perceived evaluation of children and parents across competencies.

Statements	Children	Parents
	Mean (SD)	Mean (SD)
Numeracy	3.97 (0.94)	4.07 (0.26)
Scientific literacy	4.47 (0.48)	3.83 (0.36)
Financial literacy	3.87 (0.69)	4.27 (0.31)
Cultural and civic literacy	4.03 (0.84)	3.97 (0.29)
Critical thinking / Problem-solving	4.57 (0.45)	4.30 (0.33)
Creativity	4.33 (0.47)	4.03 (0.40)
Communication	4.23 (0.57)	4.17 (0.28)
Curiosity	4.26 (0.64)	4.13 (0.32)
Initiative	4.10 (0.63)	3.97 (0.29)
Persistence / Grit	3.77 (0.67)	4.00 (0.50)
Adaptability	4.47 (0.55)	4.17 (0.32)
Leadership	4.33 (0.74)	4.20 (0.39)
Social and cultural awareness	4.30 (0.60)	4.13 (0.32)

In the broader criteria categories (Table 2), children rated GPR (Mean = 4.28, SD = 0.58) and PS (Mean = 4.25, SD = 0.39) the highest, while parents provided slightly lower ratings (GPR: Mean = 4.14, SD = 0.30; PS: Mean = 4.07, SD = 0.21). SPR received similar ratings from both groups (Children: Mean = 4.09, SD = 0.50; Parents: Mean = 4.08, SD = 0.18), indicating a shared perspective this category. The higher variability in children’s ratings suggests a wider range of individual learning experiences, whereas parents’ more consistent evaluations reflect a generalized perception of progress.

**Table 2:** Perceived evaluation of children and parents across the three criteria.

Criteria	Children Mean (SD)	Parents Mean (SD)
General progress (GPR)	4.28 (0.58)	4.14 (0.30)
Specific progress (SPR)	4.09 (0.50)	4.08 (0.18)
Physiological states (PS)	4.25 (0.39)	4.07 (0.21)

### User satisfaction assessment

Feedback on user satisfaction highlighted strengths and areas for improvement in the KideClass game (Table 3). Audio esthetics received the highest rating (Mean = 4.90, SD = 0.32), with participants praising engaging sound effects. Creative freedom (Mean = 4.65, SD = 0.67) and AR features (Mean = 4.65, SD = 0.78) followed closely, reflecting appreciation for interactive and exploratory elements. Visual esthetics were also perceived positively (Mean = 4.70, SD = 0.26), while Enjoyment scored slightly lower (Mean = 4.60, SD = 0.81).

Usability (Mean = 4.43, SD = 0.69) and narratives (Mean = 4.30, SD = 0.74) received slightly lower scores. Personal gratfication (Mean = 4.45, SD = 0.93) suggested that the game demonstrated strong educational potential. These findings underscore the game’s strengths in engaging audio-visual elements and interactivity while identifying narrative clarity and usability as key areas for enhancement.

**Table 3:** Aspects of user satisfaction assessment and their corresponding items.

Aspects	Mean (SD)	Statements	Mean (SD)
Usability	4.43 (0.69)	I know how to achieve my goals in the game.	4.4 (1.07)
		I think it is easy for me to learn how to play.	4.6 (0.70)
		I feel that the game’s timing is appropriate.	4.3 (0.82)
Narratives	4.30 (0.74)	I am excited to see where the story goes next.	4.4 (1.26)
		I feel happy when I receive all the badges.	4.3 (1.06)
		I understand the lifestyle of villagers through the gameplay.	4.2 (1.14)
Enjoyment	4.60 (0.81)	I enjoy playing the game.	4.8 (0.63)
		I am likely to recommend this game to others.	4.4 (1.07)
Creative freedom	4.65 (0.67)	I feel creative while playing the game.	4.7 (0.67)
		I can explore different aspects of the game.	4.6 (0.84)
AR features	4.65 (0.78)	I enjoy the digital models in the game.	4.7 (0.67)
		I feel excited about the realism of the digital models in the game.	4.6 (0.97)
Audio esthetics	4.90 (0.32)	I enjoy the sound effects in the game.	4.9 (0.32)
Visual esthetics	4.70 (0.26)	I enjoy the game’s graphics.	4.9 (0.32)
		I think the graphics fit the mood and style of the game.	4.5 (0.53)
Personal gratification	4.45 (0.93)	I feel successful when I overcome obstacles in the game.	4.5 (0.97)
		I find that my skills gradually improve as I overcome game challenges.	4.4 (0.97)

## Discussion

This study highlights the potential of KideClass in promoting cultural tourism and fostering 21st century skills such as critical thinking, creativity, and social awareness. Findings indicate that KideClass can enhance user engagement, knowledge acquisition, and cultural appreciation, especially among younger audiences. These results align with existing research demonstrating the effectiveness of AR serious games in educational settings^29,30^. Moreover, this research expands existing evidence by demonstrating the application of serious games in cultural heritage^21,22,31^. Notably, this study innovatively targets AR serious games to boost cultural tourism, encouraging intergenerational participation by involving children and their parents in immersive experiences at local cultural sites, such as Mahasawat Community. By embedding localized cultural narratives, the research highlights the capacity of AR games to enrich education and preserve culture, addressing methodological gaps in leveraging technology for cultural tourism.

The effectiveness of KideClass can be attributed to several key learning mechanisms. The game incorporates immersive storytelling and interactive feedback, which are foundational to constructivist and experiential learning theories^17^. By engaging users in tasks such as identifying orchid families or managing flood scenarios, KideClass fosters active participation and deeper learning. Augmented reality further enhances this process by overlaying digital elements onto real-world environments, making abstract cultural concepts interactive^32^​​. For example, users navigating a real-life lotus field while interacting with virtual guides achieve a blend of physical exploration and cognitive engagement. This dual-layered approach not only reinforces knowledge but also fosters a deeper connection with cultural heritage^33^. AR capability to integrate digital and physical realms is crucial for cultural tourism, where engagement and authenticity are paramount^34^. Thus, KideClass exemplifies how AR can bridge traditional cultural experiences with modern technological preferences.

The implications of KideClass extend beyond educational settings, offering significant benefits for cultural tourism and local community development. By engaging younger audiences in cultural exploration, the game revitalizes interest in underpromoted areas like Mahasawat Community. Its adaptability to different cultural contexts makes it a scalable tool for fostering cultural preservation^35,36^​​. Schools and tourism boards can leverage KideClass to integrate cultural education into their curricula and promotional activities. Moreover, features such as gamified challenges and interactive feedback align with modern pedagogical trends, making learning more appealing to digital natives^37–41^. The game also addresses key barriers in traditional tourism promotion, such as static presentations and limited engagement^5,6,42^. By providing a platform that is both educational and entertaining, KideClass demonstrates how AR can transform cultural tourism into a dynamic and accessible experience for diverse audiences.

User feedback indicates high levels of satisfaction with KideClass, particularly regarding its interactivity, ease of use, and engaging storytelling elements. However, there are opportunities for refinement. Enhancements could include expanding the game’s cultural scenarios to cover more diverse traditions. In addition, collaborative features could be integrated to promote teamwork^43,44^. Customizable user journeys and adaptive difficulty levels could also make the game more inclusive for varying age groups and skill levels^45,46^. Additionally, gamified rewards, such as digital badges or cultural tokens, can sustain long-term user interest^47,48^. By addressing these recommendations, KideClass and similar AR games can evolve into robust tools for both education and cultural tourism, ensuring their relevance and impact in a rapidly digitizing world.

Despite its promising results, this study has limitations that warrant further exploration. The sample size was relatively small and geographically constrained, limiting the generalizability of findings. Future research should involve diverse participant demographics to assess the feasibility of KideClass across different cultural and educational contexts. Although this study included participants aged 6–12, without further division into specific age groups, future research could explore adaptive learning pathways within the game to better tailor experiences to different developmental stages. Implementing adjustable difficulty levels or personalized learning tracks could enhance engagement and ensure that younger and older children alike benefit optimally from the game’s interactive cultural experiences. Longitudinal studies are also needed to evaluate the long-term impact of KideClass on knowledge retention and cultural appreciation. While the study highlights the development and evaluation of KideClass, the absence of comparative analysis with other traditional cultural tourism methods leaves room for further investigation. Further research should incorporate a qualitative approach to capture in-depth perspectives, providing a more understanding of how AR influences skill development and cultural tourism. By addressing these areas, future research can ensure that AR-based learning tools like KideClass reach their full potential in preserving and promoting cultural heritage.

## Conclusion

The findings of this study demonstrate that KideClass effectively engages children and their parents in both cultural exploration and the development of 21st century skills. By integrating AR with gamified learning, the game offers a novel approach to cultural tourism, making traditional experiences more appealing to younger audiences. Additionally, this AR game can promote intergenerational engagement, encouraging shared cultural experiences between children and parents. While the game’s strengths lie in its interactivity and immersive nature, improvements in usability and narrative clarity could enhance user experience and educational outcomes. Future iterations of the game could expand its cultural scenarios and incorporate features to further strengthen its role in preserving and promoting cultural heritage across generations.

## Electronic supplementary material

Below is the link to the electronic supplementary material.

Supplementary Material 1

Supplementary Material 2

Supplementary Material 3

Supplementary Material 4 

## Data Availability

The data that support the findings of this study are available from the corresponding author, up-on reasonable request. The data are not publicly available due to information that could compromise the privacy of research participants.
