# Supplementary material for: Development and evaluation of an augmented reality serious game to enhance 21st century skills in cultural tourism
Source: Sci Rep. 2025 Apr 18;15:13492. doi: 10.1038/s41598-025-95615-5 (PMC12008235; doi:10.1038/s41598-025-95615-5)
Supplement: Supplementary file 1 — Supplementary Material 1 [file 41598_2025_95615_MOESM1_ESM.pdf]

Supplementary Material 1: Descriptions of KideClass mini-games

The KideClass includes six engaging mini-games, each designed to integrate seamlessly with real-world environments and enhance various 21st century skills such as critical thinking, creativity, and scientific literacy. Below are the detailed descriptions of each mini-game, highlighting their objectives, gameplay mechanics, and educational outcomes.

---

1. Orchid Mystery Game

This game invites players to explore an orchid farm using an AR camera to scan QR codes attached to orchid plants. Each QR code reveals detailed information about orchid families, cultivation methods, and their ecological significance. Players must correctly identify the orchids and answer related questions to earn curiosity points. Rare orchids provide higher rewards, fostering persistence and scientific literacy. The game employs a star-based scoring system to encourage replayability and improvement, while badges recognize players who demonstrate mastery of specific skills. By integrating cultural and botanical knowledge, this game fosters active engagement and curiosity about ecological systems.

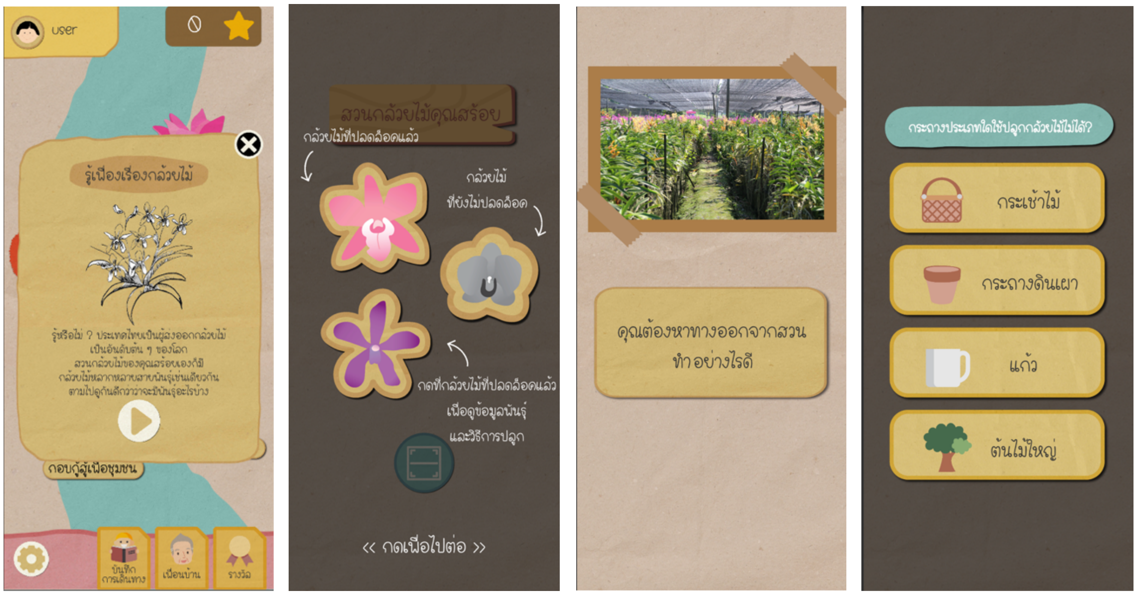

---

2. Lotus Protector Game

This game immerses players in the ecological and cultural significance of lotus farming. Using AR, players identify pests and beneficial animals that affect lotus growth. Harmful pests must be eliminated within a 30-second timeframe, and players then complete a matching game to solidify their understanding of the lotus life cycle. The gameplay develops scientific literacy by emphasizing environmental balance and cultural literacy through traditional farming practices. Stars reward performance, while badges are earned by applying knowledge effectively. This game links theoretical concepts with practical applications, creating an engaging platform for environmental and cultural education.

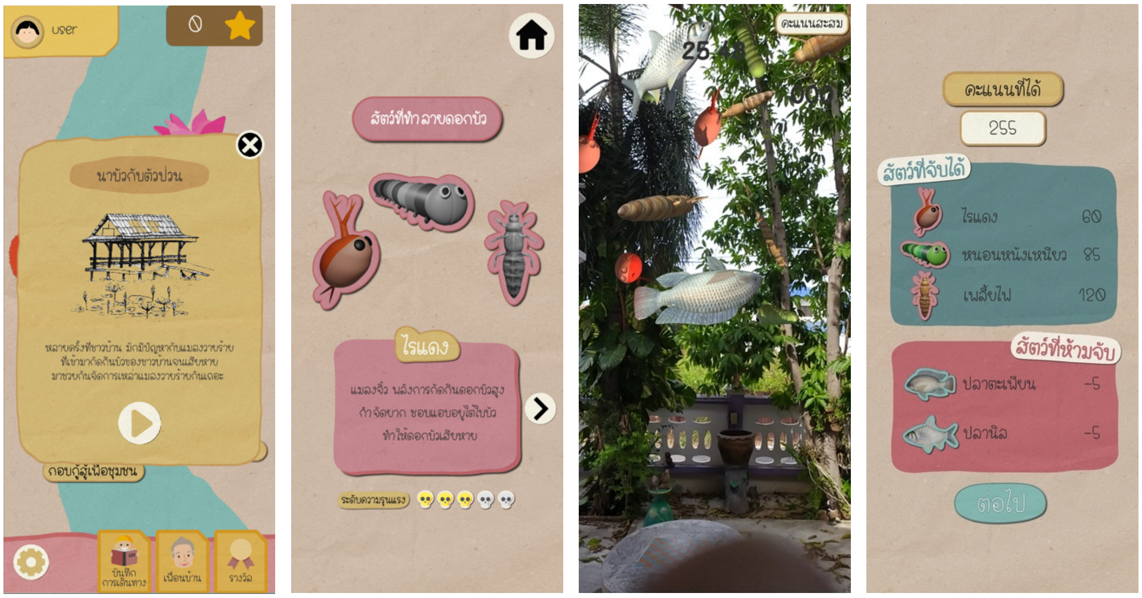

3. Fried Rice Challenge

Set in a culinary context, this game tasks players with assisting Uncle Tu in recreating his lost fried rice recipe. Players progress through interactive steps such as kneading, rolling, drying, and frying rice. The final dish must be decorated, allowing players to showcase their creativity. The gameplay emphasizes critical thinking through problem-solving tasks that require decision-making and careful analysis of instructions. Originality is rewarded with creativity points, and stars and badges recognize achievements. By connecting players to traditional culinary practices, this game combines cultural appreciation with the development of problem-solving and inventive skills.

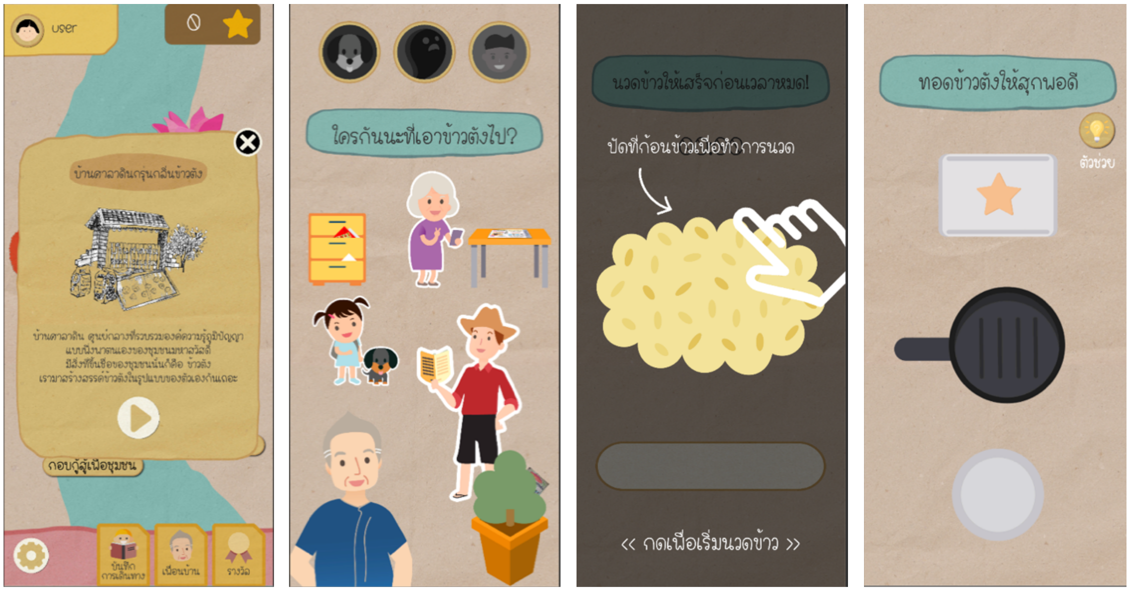

4. Gac Innovator Game

This game challenges players to address food waste by transforming surplus Gac fruit into innovative products such as juice, soap, or lotion. Using AR, players experiment with combinations of ingredients to create unique items. The gameplay fosters curiosity and initiative by encouraging exploration and testing of various ideas. Originality is rewarded, and the game highlights the economic and nutritional potential of Gac fruit. Stars and badges provide incentives for creativity and sustainability-focused problem-solving. This game promotes innovative thinking while raising awareness about resource management and the importance of sustainability.

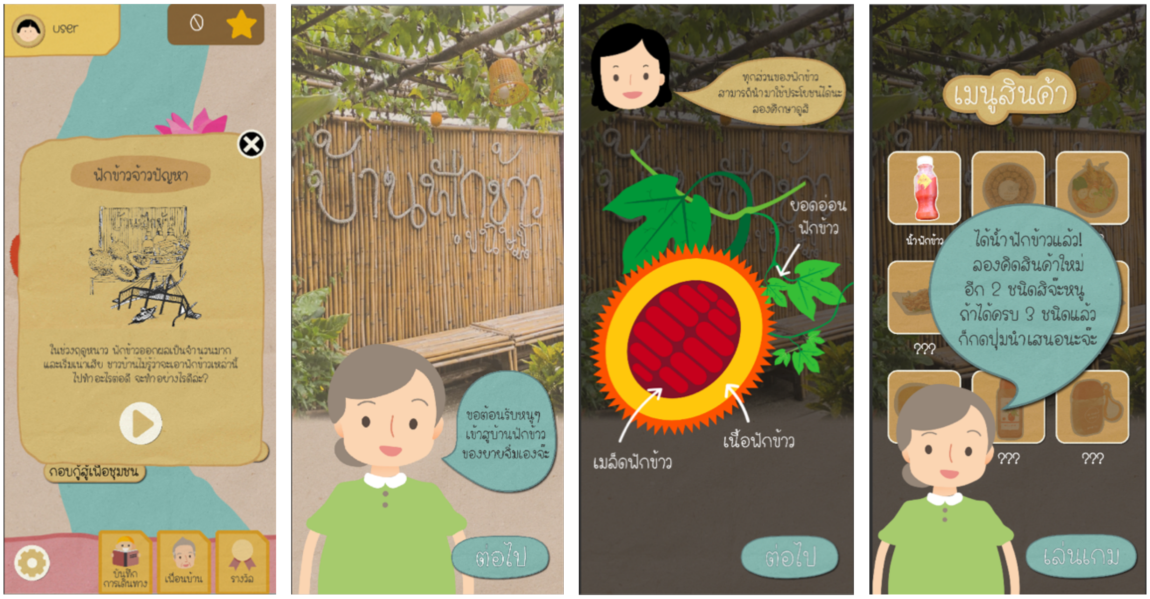

5. Fruit Harvest Adventure

This game simulates a fruit harvesting experience in collaboration with a virtual guide, Pah-Jaew. Players use an AR camera to identify and collect specific fruits within a time limit. After harvesting, they engage in real-world mathematical tasks, such as calculating prices and making correct change for virtual customers. This game strengthens numeracy through arithmetic problem-solving and enhances communication skills by requiring interaction with the guide. Stars and badges reward accuracy and efficiency, making the game a practical and engaging blend of agricultural knowledge and mathematical skills.

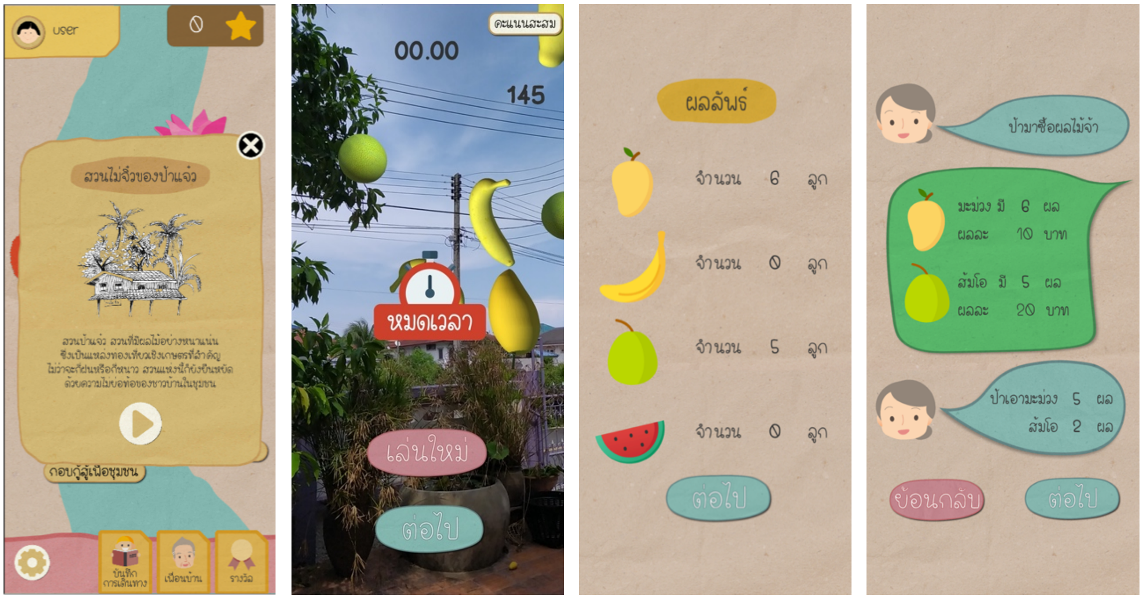

6. Community Resilience Planner

As the final and most integrative scenario, this game simulates flood management in Mahasawat Village. Players use stars earned in previous mini-games as currency to purchase resources and plan strategies across three phases: prevention, crisis management, and recovery. Players allocate resources, make leadership decisions, and support community recovery efforts, developing skills in financial literacy, critical thinking, adaptability, and leadership. Tasks such as rescuing villagers and managing flood damage test social awareness and strategic planning. Stars and badges reward successful execution, ensuring a comprehensive learning experience that ties together cultural knowledge and practical competencies.

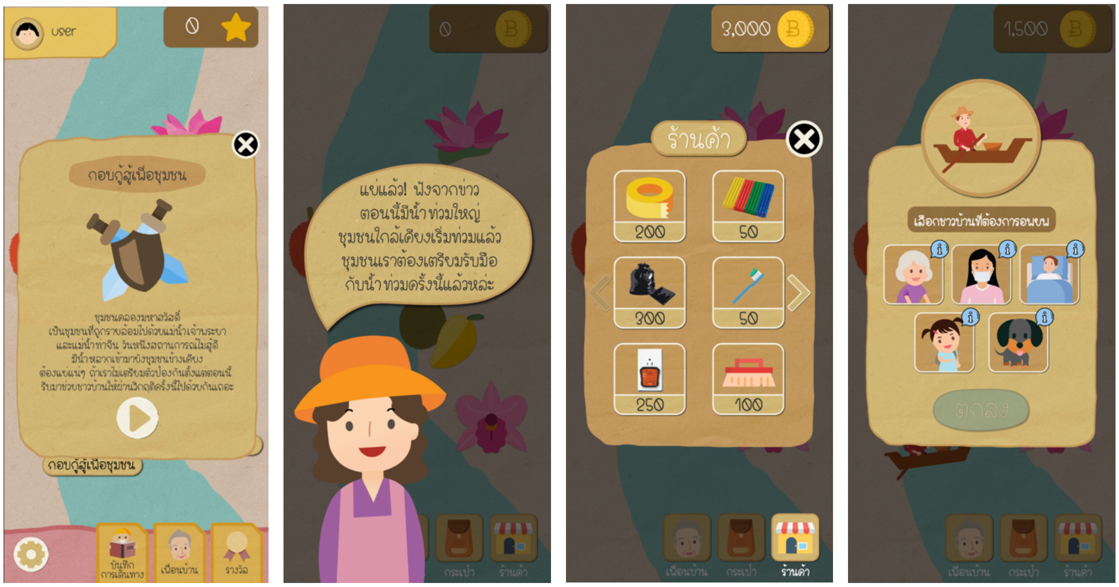

Summary

The KideClass mini-games collectively provide a robust platform for cultural and scientific education. By blending immersive AR technology with real-world applications, these games promote skill development, cultural appreciation, and environmental awareness, creating a meaningful and engaging educational experience for players.
