# Supplementary material for: Development and evaluation of an augmented reality serious game to enhance 21st century skills in cultural tourism
Source: Sci Rep. 2025 Apr 18;15:13492. doi: 10.1038/s41598-025-95615-5 (PMC12008235; doi:10.1038/s41598-025-95615-5)
Supplement: Supplementary file 2 — Supplementary Material 2 [file 41598_2025_95615_MOESM2_ESM.pdf]

Supplementary Material 2: Competencies and their corresponding items for perceived evaluation of 21st century skill development

Competencies	Statements	Criteria
Numeracy	I think math is easier for me now than before.	GPR
	I can add and subtract faster than before.	SPR
	I feel happy when I solve math problems.	PS
Scientific Literacy	I know more about science now.	GPR
	I can plant and take care of orchids by myself.	SPR
	I feel excited to learn about plants and animals.	PS
Financial Literacy	I am better at managing money now.	GPR
	I make better choices before buying things.	SPR
	I feel proud when I use money wisely.	PS
Cultural and civic literacy	I understand people's roles in the community better.	GPR
	I know more about my responsibilities.	SPR
	I feel excited to learn about how the community works.	PS
Critical Thinking / Problem-Solving	I am better at solving problems now.	GPR
	I can solve problems in the right way.	SPR
	I feel confident when solving problems.	PS
Creativity	I can come up with new ideas better than before.	GPR
	I can create more interesting things than before.	SPR
	I feel happy when I think of new ideas.	PS
Communication	I think I am more confident talking to others now.	GPR
	I can start asking good questions.	SPR
	I feel good when I talk to others.	PS
Curiosity	I think I am more curious than before.	GPR
	I want to know what happens when I explore new things.	SPR
	I feel happy when I ask questions.	PS
Initiative	I have more new ideas than before.	GPR
	I can use my ideas to do real things.	SPR
	I feel proud when I make or create something new.	PS
Persistence / Grit	I think I try harder to finish my work now.	GPR
	I can stay patient with tasks until I finish them.	SPR
	I feel happy when I finish my work.	PS
Adaptability	I understand new things better than before.	GPR
	I can find ways to handle new situations.	SPR
	I feel comfortable when I deal with changes.	PS
Leadership	I can be a leader.	GPR

Competencies	Statements	Criteria
	I can lead and take care of my friends.	SPR
	I feel happy when I am a leader.	PS
Social and cultural awareness	I understand how people in the community depend on each other better.	GPR
	I think I am ready to accept changes in the community.	SPR
	I feel kind and caring toward others.	PS
