# Supplementary material for: Development and evaluation of an augmented reality serious game to enhance 21st century skills in cultural tourism
Source: Sci Rep. 2025 Apr 18;15:13492. doi: 10.1038/s41598-025-95615-5 (PMC12008235; doi:10.1038/s41598-025-95615-5)
Supplement: Supplementary file 3 — Supplementary Material 3 [file 41598_2025_95615_MOESM3_ESM.pdf]

Supplementary Material 3: Aspects and their corresponding items for user satisfaction assessment

Aspects	Statements
Usability	I know how to achieve my goals in the game.
	I think it is easy for me to learn how to play.
	I feel that the game's timing is appropriate.
Narratives	I am excited to see where the story goes next.
	I feel happy when I receive all the badges.
	I understand the lifestyle of villagers through the gameplay.
Enjoyment	I enjoy playing the game.
	I am likely to recommend this game to others.
Creative Freedom	I feel creative while playing the game.
	I can explore different aspects of the game.
AR features	I enjoy the digital models in the game.
	I feel excited about the realism of the digital models in the game.
Audio esthetics	I enjoy the sound effects in the game.
Visual esthetics	I enjoy the game's graphics.
	I think the graphics fit the mood and style of the game.
Personal Gratification	I feel successful when I overcome obstacles in the game.
	I find that my skills gradually improve as I overcome game challenges.
