# Supplementary material for: Development and evaluation of an augmented reality serious game to enhance 21st century skills in cultural tourism
Source: Sci Rep. 2025 Apr 18;15:13492. doi: 10.1038/s41598-025-95615-5 (PMC12008235; doi:10.1038/s41598-025-95615-5)
Supplement: Supplementary file 4 — Supplementary Material 4 [file 41598_2025_95615_MOESM4_ESM.pdf]

Supplementary Material 4: Data of perceived evaluation of 21st century skill development

Statements	Criteria	Children		Parents	
		Mean	SD	Mean	SD
Numeracy		**3.97**	**0.94**	**4.07**	**0.26**
I think math is easier for me now than before.	GPR	4.1	0.88	4.4	0.52
I can add and subtract faster than before.	SPR	3.7	1.25	3.6	0.52
I feel happy when I solve math problems.	PS	4.1	1.20	4.2	0.42
Scientific Literacy		**4.47**	**0.48**	**3.83**	**0.36**
I know more about science now.	GPR	4.3	0.82	4.2	0.42
I can plant and take care of orchids by myself.	SPR	4.6	0.70	3.7	0.48
I feel excited to learn about plants and animals.	PS	4.5	0.71	3.6	0.70
Financial Literacy		**3.87**	**0.69**	**4.27**	**0.31**
I am better at managing money now.	GPR	4.0	1.41	4.3	0.48
I make better choices before buying things.	SPR	3.8	1.48	4.2	0.42
I feel proud when I use money wisely.	PS	3.8	1.23	4.3	0.48
Cultural and civic literacy		**4.03**	**0.84**	**3.97**	**0.29**
I understand people’s roles in the community better.	GPR	4.4	0.84	4.0	0.47
I know more about my responsibilities.	SPR	4.1	1.10	4.1	0.57
I feel excited to learn about how the community works.	PS	3.6	1.58	3.8	0.42
Critical Thinking / Problem-Solving		**4.57**	**0.45**	**4.30**	**0.33**
I am better at solving problems now.	GPR	4.6	0.52	4.2	0.42
I can solve problems in the right way.	SPR	4.5	0.71	4.3	0.48
I feel confident when solving problems.	PS	4.6	0.52	4.4	0.52
Creativity		**4.33**	**0.47**	**4.03**	**0.40**
I can come up with new ideas better than before.	GPR	4.4	0.84	4.1	0.32
I can create more interesting things than before.	SPR	4.3	0.95	4.1	0.32
I feel happy when I think of new ideas.	PS	4.3	0.48	3.9	0.74
Communication		**4.23**	**0.57**	**4.17**	**0.28**
I think I am more confident talking to others now.	GPR	4.3	0.82	4.3	0.48
I can start asking good questions.	SPR	3.8	1.23	4.1	0.32
I feel good when I talk to others.	PS	4.6	0.52	4.1	0.32
Curiosity		**4.26**	**0.64**	**4.13**	**0.32**
I think I am more curious than before.	GPR	4.0	1.25	4.3	0.48
I want to know what happens when I explore new things.	SPR	4.3	0.67	4.2	0.42
I feel happy when I ask questions.	PS	4.5	0.53	3.9	0.57
Initiative		**4.10**	**0.63**	**3.97**	**0.29**
I have more new ideas than before.	GPR	4.4	0.84	3.6	0.52
I can use my ideas to do real things.	SPR	4.1	1.20	4.2	0.42

Statements	Criteria	Children		Parents	
		Mean	SD	Mean	SD
I feel proud when I make or create something new.	PS	3.8	0.92	4.1	0.32
Persistence / Grit		**3.77**	**0.67**	**4.00**	**0.50**
I think I try harder to finish my work now.	GPR	4.1	1.10	3.8	0.63
I can stay patient with tasks until I finish them.	SPR	3.4	1.43	4.1	0.57
I feel happy when I finish my work.	PS	3.8	1.40	4.1	0.57
Adaptability		**4.47**	**0.55**	**4.17**	**0.32**
I understand new things better than before.	GPR	4.6	0.70	4.2	0.42
I can find ways to handle new situations.	SPR	4.0	1.05	4.1	0.57
I feel comfortable when I deal with changes.	PS	4.8	0.42	4.2	0.42
Leadership		**4.33**	**0.74**	**4.20**	**0.39**
I can be a leader.	GPR	3.9	1.45	4.1	0.57
I can lead and take care of my friends.	SPR	4.4	0.84	4.3	0.48
I feel happy when I am a leader.	PS	4.7	0.67	4.2	0.63
Social and cultural awareness		**4.30**	**0.60**	**4.13**	**0.32**
I understand how people in the community depend on each other better.	GPR	4.6	0.52	4.3	0.48
I think I am ready to accept changes in the community.	SPR	4.2	0.79	4.0	0.47
I feel kind and caring toward others.	PS	4.1	1.10	4.1	0.32
